# Acknowledgment to the Reviewers of *Biomolecules* in 2022

**DOI:** 10.3390/biom13020199

**Published:** 2023-01-19

**Authors:** Biomolecules Editorial Office

**Affiliations:** MDPI AG, St. Alban-Anlage 66, 4052 Basel, Switzerland

High-quality academic publishing is built on rigorous peer review. *Biomolecules* was able to uphold its high standards for published papers due to the outstanding efforts of our reviewers. Thanks to the efforts of our reviewers in 2022, the median time to first decision was 16 days and the median time to publication was 38 days. Regardless of whether the articles they examined were ultimately published, the editors would like to express their appreciation and thank the following reviewers for the time and dedication that they have shown *Biomolecules*:
A. A. KorzhenkovKumar KatraguntaA. K. M. Firoj MahmudKumari KavitaAabid Manzoor ShahKumiko SaekiAaron MuthKundlik GadhaveAaron OakleyKunihiro KuwajimaAaron PollettKunka Mohanram RamkumarAbbas JarrahiKun-Lin TsaiAbdelfattah M. SelimKuo Hao LeeAbdelkareem Abdall AhmedKyle S. McCommisAbdelkarim AbousalhamKyohei FurukawaAbdollah AllahverdiKyoungtae KimAbdorreza Naser-MoghadasiKyriaki KyriakidouAbeda JamadarKyung Bo KimAbel Martínez-RodrigoKyung Sik ParkAbhijit ShirkeKyung-Ah LeeAbhinav DubeyKyung-Jin MinAbhinav KaushikLaith AlzubaidiAbid H. BandayLan MaAbozar GhorbaniLarisa AnghelAbraham Cisneros-MejoradoLarissa Bueno TofaniAbraham Wall MedranoLarissa KotelevetsAdam KreutzerLasse LindahlAdam LiwoLászló DuxAdam Thean Chor LeowLászló VécseiAdam WalkowiakLaszlo VighAdetola AdesidaLatha RamireddyAdina HutanuLatif SumeraAdithya SridharLatifa ElantakAditi BhargavaLaura AlanizAditya GanjuLaura Ancuţa PopAdolfo Baloira VillarLaura BarsantiAdolfo PomaLaura BelverAdrian RothenfluhLaura BohnAdrian Velázquez-CampoyLaura GutiérrezAdriana Bos-MikichLaura M. Hunsicker-WangAdriana R. RodriguesLaura MaziluAdriana Ribeiro SilvaLaura RaimondiAdrianne Wallace-PovirkLaura RosanòAdriano AraujoLaura XicotaAdriano PiattelliLaurence LeherteAdrover MiquelLaurent MullerAftab TaiyabLaurentiu M. PopAfzal HussainLeandro CastroAgata GozdzLeandro Dias Da SilvaAgata JanowskaLee Lee WongAgnès RötigLee Wei LimÁgnes TantosLeena HaatajaAgnieszka B. BialkowskaLeena P. BharathAgnieszka Dettlaff-PokoraLei LiuAgnieszka DudziakLei LuAgnieszka FogelLei ZhangAgnieszka KrawczenkoLela BuckinghamAgnieszka KuchtaLenka RossmeislováAgnieszka ŁukomskaLeo RascheAgnieszka Łupicka-SłowikLeonard M. NeckersAgnieszka MroczekLeonardo BencivengaAgnieszka RuszkowskaLeonardo BrunettiAgnieszka SzewczykLeonardo FerreiraAgnieszka TudekLeonardo Medrano SandonasAgnieszka WaclawikLeonel PereiraAgnieszka WąsikLeonid Pavlovich ChurilovAgnieszka Zdybicka-BarabasLeonor Puchades-CarrascoAgostino MarrazzoLeopold EckhartAgostino MiglioreLeopoldo González-BrusiAgostino OgnibeneLetizia AngiolellaAgustín HernándezLetizia MattiiAhmad Pahlavan TaftiLiana A. BasovaAhmed A. Abd El-MaksodLiang HuAhmed A. ElolimyLiang MaAhmed BadrLiang ZhaoAhmed LasfarLianghui ZhangAhmed LazrakLiang-Jun YanAhmed M. DebelaLianxin LiuAhmed M. SayedLichun HeAhmed MohsenLidia CicconeAhmed RakibLidia GaffkeAhmed S. MontaserLidija GradišnikAhmed Sherif AttiaLidija TodorovicAidan Bradley GrosasLidija-Marija TumirAimin YangLijin TianAinhoa Plaza-ZabalaLijuan FengAivars VembrisLilach GavishAjaz BhatLilach SoreqAjit RayLilia Y. KucheryavykhAjit SinghLiliana Ostopovici-HalipAjmal Koya PulikkalLiming ChenAkalabya BissoyiLiming ZhangAkihiro IshiiLin HuangAkinori HaraLin SuAkira MatsumoriLin WangAkira NaitoLinda PenfoldAkira ObanaLindsay BrownAkira SatoLindsay MarshallAkitatsu HayashiLing ZhaAkito MaeshimaLingling ZhaoAklank JainLingling ZhengAkos A. GerencsérLingwen DingAlain SarauxLinus O. JohannissenAlan KaplanLionel BallutAlan ReinLiqing ZangAlan WuLiqun LiuAlarico ArianiLisa Harrison-BernardAlbert GuskovLisa-Maria NeedhamAlberto Fernández-MedardeLisanework AyalewAlberto Jorge Oliveira LopesLiubov BuchynskaAlberto Marin-SanguinoLiubov GorbachevaAlbina TummoloLiudmila A. AlexandrovaAlbino CarrizzoLivnat Afriat-JurnouAldo Ferreira-HermosilloLiz SimonAlejandro GugliucciLjubisa NikolicAlejandro Schcolnik-CabreraLoïc DayonAleksandar Ž. KostićLoic LemonnierAleksandra Elzbieta Badaczewska-DawidLong HanAleksandra Hecel-CzaplickaLong YuanAleksandra Karczmarska-WódzkaLorand KissAleksandra M. BondžićLoredana CapobiancoAleksandra MarciniakLorella MarselliAleksandra NešićLoren B. AndreasAleksandra Piechota-PolanczykLorena UrbanelliAleksey ChaulinLorenz AdlungAleksey M. VishnyakovLorenza VantaggiatoAleksey ZaitsevLorenzo GoliniAlessandra CarèLorenzo NestiAlessandra Fiorio PlaLorenzo PolimenoAlessandra Maria VitaleLothar BreckerAlessandra PolissiLouis-Charles BélandAlessandra SinopoliLouise ReillyAlessandra StacchiottiLu XiaoAlessandra TessitoreLu Yao WeiAlessandro AlaimoLu ZhengAlessandro GranitoLubecka KatarzynaAlessandro MarroneĽubomíra TóthováAlessandro MartoranaLuc BoussetAlessandro MatteLuca AmbrosioAlessandro PaiardiniLuca CapriottiAlessandro ParodiLuca FontanesiAlessandro PoggiLuca FrattaruoloAlessandro SammarcoLuca GallelliAlessia ArcaroLuca MastorinoAlessia GambadoroLuca Oscar Redaelli De ZinisAlessia RemiganteLuca PalazzoloAlessio Filippo PeritoreLuca SementaAlessio MengaLuca SimulaAlex BottLuca VanellaAlex GregorieffLucas Miranda KangussuAlexander BorodavkaLucas Opazo-RiosAlexander I. AlexandrovLucia A. SealeAlexander MatyushenkoLucia SanjurjoAlexander PanovLucia ScisciolaAlexander SapozhnikovLuciana Oliveira AlmeidaAlexander SmithLuciana Pereira RangelAlexander SuvorovLuciana ScottiAlexander TyshkovskiyLucie BrulíkováAlexander V. ProninLucimara Gaziola L. De La TorreAlexander V. VeselovskyLucinda V. ReisAlexandra A. PopovaLucio Della GuardiaAlexandra BelayewLucjusz ZaprutkoAlexandra GyongyosiLucyna Pomierny-ChamiołoAlexandra M. SminkLudmilla Morozova-RocheAlexandra NunesLuigi CastaldoAlexandra PeregrinaLuigi CristianoAlexandra Pérez-SerraLuigi PetracconeAlexandre EscargueilLuís António Paulino PassarinhaAlexandre G. de BrevernLuis BeloAlexandre MaréchalLuis Eduardo Díaz GiménezAlexandros KatranidisLuis Enrique Arroyo-GarcíaAlexandros LaiosLuis GoyaAlexandros PapachristodoulouLuis LopezAlexandru RusuLuis Mariano EstebanAlexei F. KisselevLuis MontenegroAlexei Yu LupatovLuis O. Soto-RojasAlexey NesterenkoLuiz Eduardo Nunes FerreiraAlexey P. GalkinLuiz Gustavo De Almeida ChuffaAlexey SarapultsevLuiza O. PerucciAlexey Yu SemenovLukas D. LandeggerAlexi KissLukas PrantlAlexis BroquetLukáš ŽídekAlfons NadalLukasz S. BorowskiAlfonso Varela-LópezŁukasz SzczukowskiAlfonso ZambonLuke J. NeyAlfredo CaturanoLutz KonradAlfredo Cruz-GregorioLyndsay MurrayAlfredo Jimenez-PerezLynmarie ThompsonAlfredo MartínezLyudmila Bel’skayaAlfredo PulvirentiLyudmila I RachekAlfredo Téllez-ValenciaM. Beatriz Durán AlonsoAli BahadurM. Dolores MolinaAli ChâariM. Esther GallardoAli JazirehiM. Quadir SiddiquiAlicia G. Marroquín-CardonaM. Rezaul IslamAlicia Mercedes Viloria-PetitM. SteenmanAlicia Rodríguez-BarberoMaartje NielsenAlicia RoqueMaciej Iek SkrzypczakAlicia Sánchez-MendozaMaciej JarzebskiAlik HonigmanMădălin-Iancu EnacheAlina KuryłowiczMadduri SrinivasAlina PykaMadhavan NampoothiriAlisa SokolovskayaMadhu SinghAlkmini T. AnastasiadiMadhuvanthi VijayanAllan Bruce DownieMagalie BoguenetAllan Chris M. FerreonMagdalena A. MozolewskaAllen Sam TitusMagdalena CzemierskaAllison MackieMagdalena Jastrzębska-WięsekAllyn HowlettMagdalena KostkiewiczAlma D. Alarcon-RojoMagdalena Lebiedzinska-ArciszewskaAltaner CestmirMagdalena SastreAlvaro GlavicMagdalena WójciakÁlvaro Gutiérrez UzquizaMagdalena ŻurawekAlvaro MaciasMagdalene J. SeilerAlvaro MesoracaMaged S. Abdel-KaderAmalia Enríquez-de-SalamancaMagnus KjaergaardAman UllahMagriet A. van der NestAmanda BrinkworthMahendra SinghAmanda EvansMaher Y. AbdallaAmanda IglesiasMahesh GokaraAmarda ShehuMahi M. MohiuddinAmeer Fawad ZahoorMahmoud A. SenousyAmelia BarilliMahmoud Al-KhrasaniAmelia Franke-RadowieckaMahmoud ElfakyAmer AhmedMahmoud KandeelAmin ZakeriMai MostafaAmir DoriMaite OrruñoAmir Farokh PayamMaja MarušičAmir RostamiMaja MatulicAmit KumarMaja MatulićAmit PalMaja-Theresa DieterlenAmit SinghMakoto EndoAmitava AdhikaryMaksim RebezovAmiya PatraMalgorzata BurekAmmad Ahmad FarooqiMałgorzata CytryńskaAmnon HorovitzMałgorzata JeleńAmr GamalMalgorzata KasztanAmr NegmMałgorzata LenartowiczAmrinder SinghMałgorzata Lewandowska-SzumiełAmritlal MandalMałgorzata MarczakAmro AbulilaMamoru SatoAmy FindlayMan Sau WongAmy S. LeeManas KotepuiAna C. Martins CavacoManasi TamhankarAna Carolina Pinheiro CamposManendra Babu LankadasariAna CaruntuManikandan PanchatcharamAna CazacuManindra TiwariAna Čipak GašparovićManish KumarAna D. Popović-BijelićManish Vijaya KumarAna DascaluManjón ElviraAna García-OstaManju SharmaAna José Jiménez-AraujoMan-Kyo ChungAna Josefa SolerManlio TolomeoAna M. PelachoManohar RadhakrishnanAna Margarida AraújoMansoor HussainAna Maria da Palma MoreiraMansoore EsmailiAna María Díaz LanzaMansour SobehAna Maria TeixeiraMantas ZiaunysAna Maria UdreaManuel Almenara-BlascoAna Mendes LealManuel Contreras-LópezAna OliveiraManuel FuentesAna Podolski-RenićManuel GarrosaAna Raquel MaceirasManuel López-CabreraAna Rita Xavier De Jesus GameiroManuel MuroAna SampaioManuel PestanaAna Sofia SilvaManuela E. GomesAna Sofia VallésMar Giner-CalabuigAna Teresa ReisMara BrancaccioAna TomićMara GagliardiAnabela Bernardes da SilvaMāra PilmaneAnanda Ayyappan Jaguva VasudevanMarc C. A. StuartAnandharajan RathinasabapathyMarc Le VéeAnastasia A. AnashkinaMarc PocardAnastasia De LucaMarcel BermudezAnastasia FrolkovaMarcel SchmidtAnca-Roxana PetroviciMarcel TawkAncha BaranovaMarcela AragonAn-Chen ChangMarcello CiaccioAnders BarthMarcello MeloneAnders LarssonMarcello PintiAndras BikovMarcelo A. LimaAndrás MicsonaiMarcelo N. MuscaraAndrás MihályMarcia ManterolaAndraz DovnikMarcia RatnerAndré L. SimãoMárcia T. RodriguesAndre MenkeMarcin KucińskiAndrea Adamcakova-DoddMarcin KurekAndrea AntosovaMarcin OkrójAndrea BerenyiovaMarcin SajekAndrea BossoMarcio Argollo De MenezesAndrea C. RuthesMarco G. AlvesAndrea CanaleMarco MangiagalliAndrea CorniaMarco MazzoranaAndrea DekanicMarco MelladoAndrea GiansantiMarco MilaneseAndrea KravatsMarco SegattoAndrea PoloMarco TatulloAndrea RomaniMarcos Egea Gutiérrez-CortinesAndrea StoccoroMarcos Fernando Xisto Braga CavalcantiAndrea TangherloniMarcus GrimmAndrea VanniniMarcus HöringAndreas HochwagenMarcus LancéAndreas KanavosMardelle AtkinsAndrei LukashkinMarek K. BernardAndrei SurguchovMarek LapkaAndrej BesseMarek OrłowskiAndrej ŠušorMarek PieszkaAndrés García-GarcíaMarek TchórzewskiAndrés Velasco-VillaMaren MüllerAndrew D. RedfernMargaret WorrallAndrew J. LarnerMargarita A. GoldbergAndrew MallettMargherita CorrentiAndrew R. BondMargherita EufemiAndrew Robert BurkeMargherita GianniniAndrew SpiersMargherita MaioliAndrew T. TemplinMargherita PuppoAndrey A. GorchakovMargot ErnstAndrey BogoyavlenskiyMargot Mayer-ProschelAndrey ErkinMargreet R. De VriesAndrey MusatovMaria Agnese Della-FaziaAndrey Y. VinokurovMaría Ángela Burrell BustosAndriy E. BelevychMaria Angela LosiAndrzej BajguzMaria BovaAndrzej KloczkowskiMaria Cristina De RosaAndrzej PolańczykMaría D. CouceAndrzej SkrętMaria Elena RussoAne Murueta-GoyenaMaria Fernanda Setúbal Destro RodriguesAnel Gómez GarcíaMaria FortofoiuAng LiMaria Gabriella GabrielliÁngel CoresMaria GaczynskaAngel Josabad Alonso-CastroMaria Giovanna LupoAngela BarretoMaria Giovanna ScioliAngela CasarilMaria Grazia MurraliAngela FleischhackerMaria Helena Andrade SantanaÁngela MachucaMaria IgottiAngélica Román GuerreroMaria Isabel Ribeiro DiasAngelique Regnier-GolanovMaria Isabel VeigaAngelisa FrascaMaria João LançaAngelo BañaresMaria João LimaAngelo SpinelloMaria Jolanta RędowiczAngiola ForleoMaría José García-PolaAngus M. BrownMaria KalivaAnibal CoronelMaria LeporeAníbal De Freitas Santos JúniorMaria LittmannAnil JeggaMaria LomovaAnil TharappelMaría Lourdes AbarcaAnilkumar Thaghalli ShivannaMaría Luisa López-CastejónAnindita DasMaría Luisa MoyáAnirudh GaurMaría Luna-LunaAnis Ben HsounaMaria Madalena RinaldiAnisha ShakyaMaria Manuela GasparAnita Kloss-BrandstätterMária MastihubováAnita RywińskaMaría Moreno-del ÁlamoAnja KarlstaedtMaria MunozAnja Kathrin JaehneMaria OttAnja KolaričMaria RicciAnja PišlarMaria RicciardiAnkit GangradeMaria Rita BiancoAnkit SrivastavaMaria Rosa BecciaAnkita BurmanMaria RositoAnn HorsburghMaria Serafina RistaldiAnn Van SoomMaria TalmonAnna B. VolnovaMaria Teresa CeccheriniAnna BaranMaria V. TurovskayaAnna Benedetta Dalla PalmaMaria VaccaroAnna Bilska-WilkoszMaria WrobelAnna BirkováMaria XilouriAnna BurdzinskaMaria-Alexandra MartuAnna CampanatiMarian SzebenyiAnna Cleta CroceMariana SantosAnna D’ AlessandroMarianna BarbalinardoAnna DalinovaMarianna NalliAnna Di MatteoMariano Francesco CaratozzoloAnna Drelich-ZbrojaMariano VenanziAnna ElkinaMariapaola MarinoAnna GładyszMarie ČernáAnna HardinMarie HennebelleAnna Jamroz-WisniewskaMarie WeinhartAnna KleczkaMarie-Emilie TerretAnna Lisa GiulianiMarie-Joelle VirolleAnna MamaevaMarie-Louise ElkjaerAnna Maria D’UrsiMarie-Paule GonthierAnna Maria SantoroMariia NesterkinaAnna Maria VettrainoMarija GredicAnna Martina BattagliaMarija MeznaricAnna NowinskaMarike GeldenhuysAnna OnopiukMariko SaitoAnna RealeMarilia Oliveria Fonseca GoulartAnna RonowskaMarilyn CornelisAnna SansoneMarin MicutzAnna SchritzMarin RojeAnna SpagnolettaMarina GomzikovaAnna StasiakMarina I. GarinAnna TintiMarina QuartuAnna V. GoncharenkoMarina RomozziAnna VangoneMarina ScarpaAnna ŻbikowskaMarina SciancaleporeAnna ZemancikovaMarina SpînuAnnalisa MasiMarina V. PadkinaAnnalisa PecoraroMario AcunzoAnnamária KincsesMario CapassoAnnamaria OffidaniMario H. BarrosAnnarica CalcabriniMario Luca MorieriAnnat RaiterMario Mencia CaballeroAnne ChiaramelloMario SchubertAnne JörnsMariola Sliwinska-MossonAnne Marie LauzonMarion CurtisAnne VirsolvyMariona RiusAnne ZwartsenMarios KrokidisAnne-Claude CamprouxMarisa ShiinaAnne-Lyse DucrestMariusz HartmanAnne-Marie CaminadeMariusz SikoraAnne-Sophie WoznyMarjan VračkoAnnette BrandtMark A. WilsonAnnie Frelet-BarrandMark BerryAnnika SchmidtMark F. WiserAnoop KumarMark PostAnoop RawatMark RogersAnselm C. LeeMark S. JohnsonAnshuman MishraMark WoodfordAnthony J. BerdisMarko JukičAntionette L. WilliamsMarko KrstićAntje BanningMarko MarhlAntoine CaillonMarkus A. HartmannAnton KutikhinMarkus KowarikAnton V. NaumovMarkus NettAntonella CaniniMarkus SchoenbergAntonella ManniniMarlena Gąsior-GłogowskaAntonella MeloniMarlena ZyśkAntonella PaladinoMarlene LúcioAntonella RosiMarlin ToumaAntonella ScorzielloMarta Jóźwiak-BębenistaAntonella VimercatiMarta LualdiAntonella VirgilioMarta RoccioAntoni WiedlochaMarta Š. PrnováAntonia ReimerMarta SkowrońskaAntonia ResslerMarta WłodarczykAntonia VlahouMartin BartasAntonietta BernardoMartin BommerAntonina V. StarodubovaMartin CrespiAntonino BelfioreMartin FeulnerAntonino TuttolomondoMartin LawrenceAntonio BellastellaMartin MetzgerAntonio BiondiMartin ModrianskýAntonio Di StasiMartin PešlAntonio FacchianoMartin RasporAntónio FerreiraMartin RuthardtAntonio MacciòMartin SalzmannAntonio ManentiMartin Schmidt-HieberAntonio Pérez-PérezMartin StimpfelAntonio RizzaMartin ThurnherAntonio StarčevićMartin VögeleAntonio TrovatoMartin ZofallAntonios GiannopoulosMartina CaiazzaAntonius R. B. OlaMartina RalleAntony BraithwaiteMartina ZíkováAntreas KantarosMarvin SchulteAntu DasMarwan A. HabibaAnuar Salazar-GómezMarwan KwokAnuhya KommalapatiMary Miu Yee WayeAnupam DhasmanaMaryam DaneshpourAnupam SharmaMarzenna NasiadekAnuraag BoddupalliMarzia Bruna GariboldiAnurag MisraMarzia OgnibeneAnwar RayanMarzia PucciApor Veres-SzékelyMarzia VasarriApostolos PapachristosMasafumi MurataniArabela Elena UnteaMasahide SakabeArantza InfanteMasahiro IwamotoArasu GanesanMasahiro KatohArchana GiriMasahiro TamoiArchita Venugopal MenonMasahiro WakaoAri KoivistoMasakazu SekijimaAri MeersonMasaki HataArinze OkoliMasaki KobayashiAris TsalouchosMasanori AritaArkajyoti PaulMasao YamaguchiArlei MarciliMasaru TanakaArlyng GonzalezMasashi KawamiArmando CaseiroMasashi ToyodaArmando CeveniniMasato YasuiArmando PatrizioMasayori HagimoriArmando RojasMasayuki SatakeArmen MulkidjanianMassimiliano CadamuroArmenio BarbosaMassimiliano F. PeanaArnaud GissotMassimiliano MeliArne BöttcherMassimiliano ZaniboniArnold von EckardsteinMassimo BergerAroonsri PripremMassimo D’AgostinoÁrpád TósakiMassimo DonadelliArtem BadasyanMassimo FantiniArthur D. TinocoMassimo GrilliArti V. ShindeMassimo IacovielloArtur KasprzakMassimo MariottiArtur PałaszMassimo TommasinoArun Kumar NayanataraMassoud MirshahiArun SamiduraiMasumi EtoArunava GhoshMatas JakubauskasArup ChakrabortyMatea Nikolac PerkovicAsad JanMateusz JankowskiAsha KumariMathias MontenarhAshfaq AhmadMathur S. KannanAshis MondalMatteo AudanoAshish KabraMatteo BocciAshish Kumar AgrawalMatteo MoriAsier RuizMatthew LiptakAsker KhapchaevMatthew R. SkeltonAsli OzmenMatthias BallauffAsterios TsiftsoglouMatthias David ErlacherAstgik PetrosyanMatthias KolibabkaAsunción CarmonaMattia ChiesaAthina GeronikakiMattia QuattrocelliAtsushi FukushimaMattia VoltaAttila FarsangMaura Téllez-TéllezAudrey LafrenayeMaurício Da Silva KrauseAudrey N. ChangMauricio García MateuAurelio ScavoMaurizio NordioAuta JamesMaurizio TaurinoAvinash KadamMauro ChinappiAydi AbdelkarimMauro FasanoAydin GülsesMauro MagnaniAyelén ToroMaxim A. GureevAyslan Castro BrantMaxim MolodtsovAyush RanawadeMayuko IchimuraAzamal HusenMayur B. KaleAzamat TemerdashevMayur DokeAzhar ZamMazaher AhmadiAzzurra StefanucciMazhar HussainBabak EslamiMd Ahasan HabibBahaa ElgendyMd Ashik UllahBaiba SvalbeMd Shahid SarwarBaiba VilneMd. SharkerBalachandran ManavalanMeerambika MishraBalaji NagarajanMehboob HoqueBalakrishna KoneruMehdi MollapourBalawant KumarMehedi HasanBalázs BarnaMehmeti IlirBálint NagyMeichi ChangBanghyun LeeMeike J. SaulBao XiaopingMei-Ru ChenBaohong ZhangMengqian ChenBaohui XuMengying YangBaoqing SunMenno HoekstraBappaditya ChandraMepur RavindranathBarbara Eleni RosatoMercedes FernandezBarbara ŁapińskaMercedes Garcia-GilBarbara MalawskaMerica Glavina-DurdovBarbara MavroidiMeritxell CanalsBarbara MessnerMiao LiBarbara Michalec-WawiórkaMichael AssfalgBarbara MulloyMichael D. PurdyBarbara PicconiMichael DietrichBarbara RossiMichael G. BergBarbara Szeiffova BacovaMichael GalperinBarbora VlkovaMichael GareljaBarry MilavetzMichael GruschBarry WuMichael HackenbergBart LoeysMichael HildebrandBartłomiej MazelaMichael McMurrayBartłomiej PerekMichael MorganBartłomiej ZieniukMichael OverduinBartosz ŚwiderskiMichael PoteserBarun MaityMichael PyatibratovBashayer R. Al-MubarakMichael R. DoschakBasheer MannasahebMichael Robert HorsmanBasini GiuseppinaMichael RocheBato KoraćMichael V. UgrumovBawa PushpinderMichael WagnerBeata Hukowska-SzematowiczMichael WoeltjeBeata JabłońskaMichael X. ZhuBeata Kasztelan-SzczerbinskaMichaela RumlováBeata Morak-MłodawskaMichal GoldbergBeata Mrozikiewicz-RakowskaMichał KlimczakBeata NowakMichał NowickiBeata PajakMichał OczkowskiBeatrice MalacridaMichał ZarobkiewiczBeatriz Buentello-VolanteMichel JaquinodBeatriz Martín-AntonioMichela BertolaBehrooz H. YousefiMichela CastagnaBela GyurcsikMichela CinquiniBela PappMichele De BortoliBen A. BahrMichele Dei CasBenedetta GhezziMichele FioriniBenedict-Tilman BergerMichele GiannattasioBeniamin Oskar GrabarekMichele MaffiaBenjamín FloránMichele PerniBenjamín PinedaMichele SalleseBenjamin RollesMichelle HawkinsBenoît BédatMicholas Dean SmithBenoit GauthierMiglė BacevičienėBenoit MasquidaMiguel Ángel PrietoBenoit MichotMiguel EstravisBenoît MiottoMiguel Martin-CaraballoBerislav ZlokovicMihaela CudalbeanuBernd KammererMihaela NiculaeBerner Andrée Sandoval-RamírezMihai CenariuBertrand LiagreMihaly MezeiBertrand VilenoMikhail BlagonravovBetty Yuen-Kwan LawMikhail ChurnosovBhagyashri BurguteMikhail KhvostovBhawik Kumar JainMikhail KryuchkovBianca SclaviMikio HayashiBiao YanMikko Uusi-OukariBibekananda KarMiklós LengyelBidisha RoyMilan SenćanskiBilon KhambuMilardi DaniloBilqees BanoMilena RadunovicBingjian FengMilica M. SredojevićBingqiang LiuMilica MarkovicBinita ShresthaMilica SimovićBisei OhkawaraMilos LazarevicBiswajit PadhyMin GaoBjørnar HasselMin Tang-SchomerBlanco-Canosa Juan BautistaMindaugas LiaudanskasBo DingMinerva Codruta BadescuBo Hyon YunMing YangBo ZhaoMing-Tzu TsaiBogdan Ionel TambaMing-Yii HuangBogdan SevastreMin-Hao KuoBoleslaw T. KarwowskiMin-Huan WuBoris U. StambukMin-Hui LiBoris ZaslavskyMin-Ji KimBoya LiuMinjie ZhangBoyong KimMinoru TakasatoBożena Szyguła-JurkiewiczMintu ChandraBranka Salopek-SondiMinwei XuBrenda L. Sánchez-GaytánMiquéias Lopes-PachecoBrenda LeeMircea TampaBrendan Paul BurnsMireia AlcaldeBrent M. ZnoskoMireia Martín-SatuéBrian E. ApplegateMireia TondoBrian R. FrancisMiriam EchevarriaBrian SuttonMirjam SchuchardtBridget CarragherMirjana JerkicBrigitte HantuschMirko MaturiBrijesh SinghMirna González-GonzálezBritt-Marie SjöbergMiroslav BarančíkBruce D. UhalMiroslava KačániováBruno António CardosoMiroslava RabajdovaBruno BrandoMiroslava ZhiponovaBruno Cesar RossiniMirosław AndrusiewiczBruno FiondaMirosława Ferens-SieczkowskaBruno MelgarMitchell FaneBruno PéaultMitesh DwivediBruno Ramos-MolinaMithilesh JhaBryan Andrew KillingerMitsue NishiyamaBum-Rak ChoiMitsuru NakazawaByeong-Wook SongMiyako TanakaByung-Chang SuhMoghoofei MohsenCaixiang LiuMohamed A. El-EsawiCălin Gabriel FloareMohamed AdelCalin Mircea GhermanMohamed AliCamila Bussola TovaniMohamed AshourCamilla Albertina Dantas De LimaMohamed EgizaCamilla BeanMohamed El YamaniCara Lynne SchengrundMohamed LaradjiCarla PeregoMohamed MohanyCarla Solé CañadasMohamed Samir MohyeldinCarlo GalliMohamed YassinCarlo MorassoMohamed ZakyCarlo ReggianiMohammad AbdolahadCarlo SantambrogioMohammad Mahdi ForghanifardCarlo Travaglini-AllocatelliMohammad Muzaffar MirCarlos Alberto Brizuela RodriguezMohammad Naghavi-BehzadCarlos Alberto CañasMohammad QneibiCarlos AmeroMohammad SadraeianCarlos Guillén ViejoMohammad Usman AhmadCarlos MarcuelloMohammad Zuhaib QayyumCarlos Miguel MartoMohammad ZulkifliCarlos P. SilvaMohammed GagaouaCarlos Raéul FerreiraMohammed H. MohammedCarlos RosadoMohammed SaidCarmela RinaldiMohandoss SonaimuthuCarmelina RuggieroMohanraj RamachandranCarmelo CorsaroMohd AdnanCarmelo PugliaMohd AsgherCarmen AvaglianoMohd Shukuri Mohamad AliCarmen BurteaMohit SharmaCarmen CanavierMohsen HesamiCarole SousaMohsen Yoosefzadeh NajafabadiCarolin KolmederMoisés Rubio-OsornioCarolina Colombelli PaccaMojie DuanCarolina OteroMolishree Umesh JoshiCarrie ShawberMolly Duman-ScheelCarsten CarlbergMominur RahmanCarsten SchulteMona SaadeldinCassie S. MitchellMonica BerrondoCaterina FormichiMonica CiveraCaterina MancarellaMónica G. Mendoza-RodríguezCaterina PalleriaMonica Lisa Fernandez-QuinteroCaterina PeggionMonica MarazuelaCatherine CahillMonica SimionCatherine Faivre-SarrailhMonica YabalCatherine JaureguiMonika BorkowskaCatherine YzydorczykMonika KlimkowskaCathrin RohlederMonika LesickaCecile VindisMonika PapieżCecilia BattistelliMonika Skrzypiec-SpringCecilia GiuliviMonika TulejaCecilia PozziMonjurul HaqCélia VenturaMoon Nyeo ParkCesar Lopez-CamarilloMorena PetriniCesar SaldiasMoritz SchnelleCésar VeigaMostafa A. HussienCesare TerraccianoMotonari TsubakiChalong WachirapakornMuhamad MustafaChan ParkMuhammad NaeemChandan GoswamiMuhammad UmairChander Parkash DoraMukesh KumarChandra MadasuMukesh MeenaChandra SekaranMurali YandaChandrabose SelvarajMuruganandan ShanmugamChang GuoMurugesan ChandrasekaranChang SunMusthafa EssaChangfu YaoMuthuramalingam PandiyanChang-hyun KimMutian HuaChangLin ZhaoMutsuaki EdamaChangmei LiuMyriam LazardChangqi LiuMyunggi YiChang-Seok LeeNabeel AhmedChao WangNabendu MurmuChao ZhangNada MarićCharalampos SiristatidisNadav EladCharbel E. H. MoussaNadezhda A. GolubkinaCharith Raj Adkar-PurushothamaNadezhda N. SushchikCharles B. LovelyNadezhda V. ShilovaCharles DozoisNadine NagyCharles NortonNadja EngelCharles SchwietersNaga Venkata Sabarinath NeerukondaCharlotte JacobsenNagasuryaprasad KotikalapudiChathura SuraweeraNaglaa Fathi KhedrChaweewan JansakulNalini BoraChe-Chang ChangNanang Rudianto ArieftaCheil MoonNancy Monroy-JaramilloChen YuNaohiro YamaguchiChen-Chi WuNaoro TanakaCheng LyuNaoyuki KataokaChengfei YanNarayan ShivapurkarChenghan LiNaresh KumarChengliang WangNarsa M. ReddyChengming WangNarsimha MamidiChengpeng FanNasrollah Rezaei-GhalehChengxin ZhangNatacha IpseizCheng-Yoong PangNatalia BaulinaChengyue WuNatalia GrubaChennaiah AndeNatalia IssaevaCherie LeeNatalia López AndrésChiara BardellaNatalia NaryzhnayaChiara CastellaniNatalia V. BeloborodovaChiara CorpettiNatalia V. BelosludtsevaChiara PlatellaNatalia V. ShcherbikChiara PortaNatalia Viktorovna NizyaevaChiara VillaNatalija PolovicChia-Te LiaoNataša ŠtajnerChieh-Hsin LinNatassa PippaChien-Feng LiNathachit LimjunyawongChien-Hsing LeeNathalie Sol-FoulonChien-Te LeeNathalie Van Den BergeChih-Li LinNathan AbsalomChih-Yang WangNathan CorrellChii-Ruey TzengNathan GallantChing-Jin ChangNathan LanningChinmayi PrasannaNathan P. ManesChinmoy SarkarNatraj KrishnanChin-Mu HsuNayar SasiChintamani D. AtreyaNazmul HudaChiung-Ting WuNeda SladeChong-Tai KimNeena SinghChris MacDonaldNeha NandaChris NealeNeil P. J. PriceChrishan RamachandraNelson GomesChrishan SamuelNemany Abdelhamid Nemany HanafyChristian DrouetNemat AliChristian HulenNerea OsinaldeChristian MoritzNermi L. ParrowChristian U. OeingNesrein HashemChristian WirajaNesrine Salah Dine El-SayedChristina Anna StratopoulouNéstor Uribe-PatarroyoChristina PiperiNeus Martinez-BoschChristine AllmangNevena IlievaChristine BeuckNguyen Phuoc LongChristine C. HelmsNguyen Quoc Khanh LeChristine Delbarre-LadratNian XiongChristine E. Schaner TooleyNicholas A. SmithChristine F. WildsoetNicholas AshtonChristine HappelNicholas BradshawChristine KlemensNicholas BrownChristo KoleNicholas M. GrazianeChristof GrewerNicholas RalstonChristoph FreyerNicola ArmstrongChristophe BrunetNicola IelapiChristophe GinevraNicola SalvadoriChristopher BystroffNicolas BourmeysterChristopher C. L. WardNicolas DubuissonChristopher D. O. CooperNicolás MontalbettiChristopher P. ToselandNicolas NassarChristopher PlaisierNicolás Ramos-BerdullasChristopher S. WilliamsNicole BalascoChristopher SjowallNicole R. MurrayChristopher W. CunninghamNicoleta-Aurelia ChiraChristos K. KontosNicoletta GaglianoChuanbin YangNicolo TonaliChuancheng WuNieves PeltzerChuang-Rung ChangNihar Ranjan JenaChuanqi PengNiina BhattaraiChukwuemeka George O. Anene-NzeluNikhilesh JoardarChun GuoNikita WrightChunbin ZouNikola PopovićChunchia ChengNikolaos E PatsoukisChung-Ho E. LauNikolay V. GoncharovChunhua SongNilesh SharmaChunming YangNima RafatiChunru WangNina FilipczakChun-Song YangNina ReuvenChyong-Huey LaiNina WezynfeldCiesla MaciejNing ZhangCinzia PaganoNingning WangCiria Carolina Quintero HernándezNingning ZhaoCiro IsidoroNisansala AbeyrathnaClara IannuzziNithyananthan SubramaniyamClara PenasNitin AmdareClara PereiraNoah WeislederClaude BesmondNobuko HosokawaClaude BoucheixNobutaka NumotoClaude DidierjeanNobuyuki KimuraClaudia AndreiniNobuyuki OkahashiClaudia LombardoNobuyuki TanakaClaudia MartiniNoe Baruch TorresClaudia Muhle-GollNoelia DuarteClaudia NeunaberNoha ShawkyClaudia RossiNora MestorinoClaudia VerderioNora SzentmaryClaudia-Geanina WatzNorbert JostClaudio De LazzariNoriaki SasaiClaudio FerranteNoriaki SunagaCláudio RoqueNoriko HosoyaClaudio TaloraNoriko TakegaharaClaudio TrapellaNorio MiyoshiClaudio VelatiNorma Aidé Cortez-LemusClaudio ZuccaNorman ChiuClaudiu Ioan BuneaNoura DosokyClaudiu N. LunguNoureddine BencheikhCleber AnconiNunzio Del GaudioClément MorgatNuray AcarClément PicardOana CioancaClizia VillanoOana SandulescuColleen M. McDowellOfentse PooeČoma MatúšOikawa HiroyukiCong-Jun LiOksana LockridgeConsuelo CelestiOksana SorokinaCoralie Di ScalaOla HadadehCorinne BaratOlaf LarsenCornelia BalaOleg KovtunCornelis Johannes Forrindinis Van NoordenOleg PalyginCornelius KraselOleg SerovCorrado AngeliniOlesia MakhutovaCosimo LigorioOlga AfanasievaCosimo SpertiOlga AnatskayaCosmin PuiaOlga CherkasovaCraig HodgesOlga G. ZatsepinaCraig J. GoergenOlga GolubnitschajaCraig UlrichOlga GordeevaCrismita DmelloOlga LebedevaCristal ZunigaOlga MalyshevaCristian PeptuOlga SelyutinaCristiana GonçalvesOlga TsivilevaCristiane AguiarOlga V. MorozovaCristiano DiasOlga VaginCristina AndreaniOlga VinogradovaCristina BaraleOliver Heinrich FelthausCristina LópezOliver ThomasCristina Pinedo-RivillaOliver TrappCristina SobacchiOliver ZerbeCrystal S. ConnOlivier BarisCrystel BonnetOlivier BraissantCsaba KerepesiOliviero CarugoCynthia HaseltineOliviero GobboCyrus MotamedOlli-Pekka SmolanderD. Travis GallagherOmar N.A. DemerdashDafne Campigli Di GiammartinoOnofrio LaselvaDagmar RiemannOrawan WongmekiatDahn ClemensOsamu KumanoDaishi YamakawaOscar A. Lenis-RojasDaisuke TakahashiOscar CasisDalia AlansaryÓscar Rapado-GonzálezDa-Liang OuOurania E. TsitsilonisDalibor KodrikOuti Salo-AhenDamián Álvarez PaggiOvidiu Cristian OpreaDamian KaweckiOvidiu MituDamien ArnoultPablo ChagasDamien S. K. SamwaysPablo FonsecaDan CanaaniPablo NakagawaDan ChenPádraig D’arcyDan TesloianuPalaniyandi KaruppaiyaDana EksteinPan ZhengDan-Bogdan NavolanPanagiotis KarakaidosDandan YangPanagiotis MoulosDanial RoshandelPaniz JasbiDaniel BaeckerPaola CavallaDaniel Dalla TorrePaola GargiuloDaniel F. LeglerPaola LenziDaniel J. KellyPaola LondeiDaniel J. LewPaola PierobonDaniel JancuraPaola TucciDaniel KronenbergPaola ViganòDaniel L. Z. CaetanoPaolo ArosioDaniel OryPaolo BorattoDaniel P. CardinaliPaolo CapparèDaniel P. PotaczekPaolo CavorettoDaniel RodriguezPaolo ConflittiDaniel Romaus-SanjurjoPaolo Emilio AlboiniDaniel T. Baptista-HonPaolo FagoneDaniel WeberPaolo MeneguzzoDaniela BuchaimPaolo RuzzaDaniela CarbonePaolo ZardiDaniela D’AngeloParagi GáborDaniela DamianiParisa YousefpourDaniela GiulianiParmjit S. JatDaniela ImpellizzeriParul GuptaDaniela LeccaPascual García-PérezDaniela Maria TanasePaško KonjevodaDaniela MennerichPasqualino SirignanoDaniela Valdez-JassoPatrice DebréDaniela ValentiPatricia A. J. MullerDaniela VitucciPatricia SilveyraDaniele BelvisiPatrick CunninghamDaniele Dell’OrcoPatrick ForterreDaniele MercatelliPatrick FrantomDanijela BarićPatrick KehoeDanilo MilardiPatrick LüningschrörDanival José De SouzaPatrick SchussDanka BukvickiPatrizia PignattiDaria A. BelinskaiaPatrizia PorcuDariga AkashevaPatrycja KleczkowskaDarío Mendez-CuadroPau Loke ShowDario MizrachiPaudel YamDariusz KotlęgaPaul A. BrownDarshan KelleyPaul AirsDarya V. TeleginaPaul Charles WhitfordDat HaPaul ClaytonDavid Araújo-VilarPaul CopelandDavid CoelhoPaul GissenDavid ColemanPaul Le TurnierDavid ConversiPaul M.P. van Bergen En HenegouwenDavid DominguezPaul T. ThevenotDavid Jesse SanchezPaula LaranjeiraDavid N. DanforthPaula OssowiczDavid Ortiz De ZáratePaulina AdamskaDavid RamonetPauline Po Yee LuiDavid ŠilhaPaulo BettencourtDavid TetardPaulo Jorge GavaiaDavid V. HansenPaulo PalmaDavid VermijlenPaulo SantosDavid W. ScovillePaulo Victor Sgobbi De SouzaDavid ZemanPavan Kumar DhanyamrajuDavide CostaPavel A. ZykinDavide LauroPavel B. DrašarDavide SchiroliPavel LobachevskyDavit A. PotoyanPavel SolopovDawid StefaniukPavel ZhabyeyevDebajit DeyPavol KristianDebananda GogoiPaweł GaćDebasish Kumar DeyPawel KafarskiDebipreeta BhowmikPawel KrupaDébora FerreiraPaweł KrzyżekDeborah Pinson HyinkPawel LipinskiDeepak KumarPaweł OlczykDejun ZhangPaweł ŚwitDelia TitPayel DattaDelilah F. WoodPedro A. Jiménez GómezDenica BlazhevaPedro BrugadaDenis S. KudryavtsevPedro BullonDenise ToscaniPeep PalumaaDennis R. GraysonPeiguo YuanDeokho LeePeng LiDerek J. B. KleinveldPengfei DingDerek LoganPengxiang ZhuDerek S. BoeldtPersio Dello SbarbaDerek WilkinsonPete N. MonkDer-Yuan ChenPeter BaiDespina A. TataPeter FrykholmDevi AtukorallayaPeter HerrlichDhananjay YadavPeter J. WatkinsDhanasekaran SugapriyaPeter LittleDharmendra ChoudharyPeter LuntDhiraj P. MuralePeter MichielsenDi LiuPeter PolčicDiana Angelika OlszewskaPeter R. FlattDiana AntalPeter StärkelDiana DonatellaPetra ImhofDiana G. Zarate-TriviñoPetra PallagiDiana Hernandez-RomeroPetronella KettunenDiana Panayotova-DimitrovaPezhman MahmoodiDiego A. RojasPhilip C. BigginDiego CotellaPhilip CohenDiego Franco JaimePhilip SeiblerDiego La MendolaPhilip W. WertzDiego MoralesPhilippe A. GrangeDiego PeroniPhilippe De DeurwaerdereDiego QuirogaPhilippe FortDiego RaimondoPhilippe PourquierDiego SbardellaPhiwayinkosi V. DludlaDigant NayakPier Luigi AntignaniDikran TsitsekianPier MartelliDimitra BourbouliaPierangela TottaDimitri PoddighePierre BredelouxDimitris LambrouPierre CornelisDimitris MossialosPierre De RossiDina K. GaynullinaPierre Fernand NeuenschwanderDinesh RokayaPietro MazzeoDiogo Rodrigo Magalhaes MoreiraPietro ScicchitanoDipankar RoyPietro VeglianeseDipti KanabarPilar CossioDmitriy AtochinPilar SandovalDmitriy BogolyubovPinaki P. MisraDmitry A. ShulgaPing K. YipDmitry A. TikhonovPingwen XuDmitry AmininPingyue PanDmitry FrankPio Maria FurneriDmitry KarpovPiotr CeranowiczDmitry NamgaladzePiotr DomaradzkiDoan Duy Hai TranPiotr K. KrajewskiDoina A. TodeaPiotr MakDomenico Azarnia TehranPiotr MuchaDomenico De BerardisPolonowita AjithDomenico LioPołoński TadeuszDomenico MattoscioPontus GourdonDomenico Maurizio ToraldoPooja JadiyaDomenico MontesanoPoornananda M. NaikDomenico PalumboPrabu GnanasekaranDominic StandingPraddep Kumar BollaDominik KoszelewskiPradeep ChopraDominik S. SkibaPranabananda DuttaDominika GuzekPrasanna VaddiDominika MalińskaPrashant KumarDominique PadovaniPrasun KumarDomna KaragogeosPratibha KumariDonald CameronPratyusha MandalDonato D’AgostinoPraveen KogantiDong HanPraveen Kumar Raj KumarDong Keon YonPrem Kumar GovindappaDong Soo YangPrema Latha MallipeddiDongbao ChenPremila LeiphrakpamDong-Liang HuPritam GangulyDongliang QiuPriyamvada M. PitaleDongwoo LimPrunier ChloéDon-Kyu KimPrzemyslaw ChmielewskiDoo-Sup ChoiPuja KumariDoreen DobritzschPunit PrasadDoreen PalsgrovePuran S. BoraDoriane TrompierPurushoth EthirajDorota GałkowskaPushpendra SinghDorota WłogaPuy GarrastachuDragana NikitovicQamar U. ZamanDragica SelakovicQazi Mohammad Sajid JamalDrago BesloQi GaoDragos CretoiuQi ZhaoDrenka TrivanovicQian LiDurga Kalyani PaturiQian LiuDurga Praveen MekaQianbin ZhangE. Mark WilliamsQianfen WanE.V. SchmalhausenQiang FuEberhard VoitQiang ZhangEbony MonsonQianwei ChenEdio MaldonadoQinfang LiuEdit CsapoQing DengEdivaldo Herculano Correa de OliveiraQing WangEdmundo Bonilla GonzálezQingjun PanEdoardo BraunerQingyan XuEdoardo MilanettiQingyu ZhouEduardo Arroyo PardoQingzhong XiaoEduardo Valencia CanteroQinyu SunEduardo Zatarain NicolasQiong ZhangEdward J. VigmondQiu WuEdward John SayersQiu YinjieEdward PlowQiuwang ZhangEdward RenQonita Kurnia AnjaniEdyta Marta BorkowskaQuanah J. HudsonEfstathia K. KapsogeorgouQuentin PerraudEgoitz AstigarragaQun TangEgor OsidakQutuba KarwiEhab AbourashedR. M. LozanoEhsan Nazemalhosseini MojaradR. N. V. Krishna DeepakEhsanul Hoque ApuRabiul IslamEiji YoshiharaRachael RichardsonEileen RoulisRadim NenckaEirini GrapsaRadka VaclavikovaEkaitz Errasti-MurugarrenRadomir JasinskiEkaterina OrlovaRadomir SavićEkaterina RudnitskayaRadoslaw MaksymEkaterina Stepanova-KhramtsovaRadu C. RacovitaElaine ChowRafael A. GarduñoElda HegmannRafael Yutaka KuradomiElena ChikhirzhinaRafal BartoszewskiElena D. BazhanovaRaffaele SaladinoElena Gallo MacfarlaneRaffaele SerraElena KaschinaRaffaella BrugnoniElena LeshchenkoRaghunath SinghElena Maria VaroniRahmad AkbarElena ParmigianiRaj KumarElena PiniRaj Paul KapurElena SaccoRaja Gopal Reddy MooliElena ShashovaRajamma MathewElena TchetinaRajan ChoudharyElena VeronesiRajanikanth VangipurapuEleni RebelosRajesh ThimmulappaEleonora CianfloneRaji Rajesh LeninEli DraizenRajib SenguptaElia BariRajneesh SrivastavaElia RanzatoRaju Senthil KumarEliana ScemeRakesh S. LaishramEliane NoronhaRalf AdamElias A. KouroumalisRalph FingerhutElias ChristoforidesRamachandran PrakasamElias T. ZambidisRamesh KumarElikaee SamiraRamesh S. YadavaElisa BelluzziRamon CrehuetElisa OltraRamón Sotomayor-ZárateElisabeth JacobsenRamona DanacElisabeth PinartRandolph D. GlickmanÉlisabeth TraiffortRandy CowlingElisabetta CaiazzoRanhua XiongElisabetta MurruRanjith KamityElizabeth M. MartinRaphael CiumanElizabeth SokolRaquel De L. C. GiordanoElke AlbrechtRashid MirEllen H. KangRashmi PrasadElmer MauritsRaúl Alberto Reyes-VillagranaEloi Franco-TrepatRaúl Cazorla-LunaElsherbiny ElsherbinyRaúl Colorado-PeraltaElvira E. ShultsRavi B. ChalamalasettyElżbieta ŻbikowskaRavi Kumar SharmaEman MehannaRavi SonaniEman Y. GoharRavindra KumarEmanuel Hernández-NúñezRavindra ThakkarEmanuel WeitschekRay LuoEmanuela BostjancicRaymond BlindEmil RudolfRaymond J OwensEmiliano Raúl DiezRaymond M. QuockEmilio CarboneRebecca A. SagerEmma SolaRédouane AherrahrouEmmanuel J FavaloroReinaldo Dos SantosEmmanuel M. PapamichaelReiner SchneiderEmmanuelle BourneufReinhard DechantEmmanuelle SchmittReinhard DeppingEmöke ŠteňováReinhard LängerEmrah NikerelRekha C. PatelEncarnación Núñez OliveraRémi LonguespéeEndika Prieto-FernándezRemigiusz BąchorEnilze M. S. F. RibeiroRenan FalcioniEnoch A. MensahRenata CiccarelliEnquan XuRenata MikstackaEnrico GarattiniRenata Novak KujundžićEnrico RaveraRenata PerlikowskaEnrique MarcosRenato FerrazEnrique Morales-ÁvilaRenato HeidorEnzo LalliRené BuchetEran PerlsonRene HuberEri S. SrivatsanRene Van Der VlugtEric Boue-GrabotRenlei JiEric GhigoReza KhayatEric HajduchRicardo Franco-DuarteEric HuetRicardo LamyEric JohnsonRicardo Massarico SerafimÉric LacazetteRicardo N. PereiraErica BuosoRiccardo MassonErica Elisa FerrandiRiccardo PanellaErik Alpizar-ReyesRichard BinghamErik BehringerRichard D. CummingsErik D. HolmstromRichard GuyerErik SchoenmakersRichard HaynesErika Bevilaqua RangelRichard SindenErika PalmieriRichard W. NaylorErika StaudacherRick JansenErin G. Reed-GeaghanRick M. MaizelsErnestina Marianna De FrancescoRifat Ullah KhanErnesto García-PinedaRikki CorniolaErnesto J. FuentesRita ChenEslam M. Abdel-SalamRita FerreiraEsmaeel SharifiRita MotaEstanis NavarroRitesh SevalkarEstella MusacchioRittu HingoraniEsther HeidRobert B. RuckerEugene V. KorotkovRobert C. KrencikEugenia PechkovaRobert KammererEugenio Velasco OrtegaRobert KyptaEun Jeong ParkRobert LenkinskiEun-Taex OhRobert P. SheridanEunyoung TakRobert PasławskiEusebio ChiefariRobert PodlasekEva CoreyRobert Steven EsworthyEva KatonaRobert W. JackmanEva KnuplezRobert WeinzierlEva KostakovaRobert WessellsEva TvrdáRobert Y.S. ChengEvangelia ChronopoulouRoberta CascellaEvangelia EmmanouilidouRoberta D’Emmanuele Di VillabiancaEvelyne ManetRoberta GhidoniEvren Önay UçarRoberta PalmourEwa BalcerczakRoberta PierattelliEwa JówkoRoberto CaboEwa Kijeńska-GawrońskaRoberto CampagnaEwa Malecka-PanasRoberto CannataroEwa Olchowik-GrabarekRoberto FrauF. Luis González FlechaRoberto GualtieriFabian Den BraveRoberto La RoccaFabiana GeraciRoberto Quezada-CalvilloFabiana LucàRobertus NelissenFabio BrambillaRocco CancelliereFábio Filipe Fereira GarrudoRochelle TixeiraFabio FumagalliRodica BalasaFabio TrovatoRodica OlarFabrizio MatteiRodion J. MolotkovskyFaezeh VakhshitehRodrigo Ligabue-BraunFahad AsmiRodrigo OchoaFahad UsmanRodrigues Chaves AndreaFahmi ZairiRoger JacobsFakhrossadat MohammadiRogerio R. Sotelo-MundoFanbo MengRogério RodriguesFang LeiRoland BückerFangfang ZhongRoland G. HuberFarhad HossainRoland HartmannFarhan ChowdhuryRoland KerpesFarshad SohbatzadehRoman MatějkaFarzad SalehpourRoman P. JakobFarzaneh KordbachehRomanas ChaleckisFatih DemirRomeo T. CristinaFederica BonoRon UngerFederica D’AmicoRonald Allan PanganibanFederica De CastroRonald ChandlerFederica ManninoRonald GarnerFederica ReyRonald J. WongFederico FerroRonald NelsonFederico IovinoRonan MurphyFederico Maria RubinoRong FangFederico PalazzettiRong LiuFederico RavaioliRory Cristiane Fortes De BritoFelix ClanchyRosa A. SicilianoFelix GonçalvesRosa López-PedrajasFélix MachínRosa Maria Mateos BernalFeng JianfangRosa SacedónFeng LiRosalia CrupiFeng WangRosalia Maria Cristina PellitteriFengchao LangRosana RibićFengna LiRosanna Di PaolaFerdinand AlthammerRosaria MeccarielloFerdinando Di CuntoRosario González-MuñizFerdinando PalmieriRosario SarabiaFerdinando PaternostroRoseline MazetFerdy R. Van DiemenRosemary J. AkhurstFerenc A. AntoniRossana SolettiFerenc SiposRossella MieleFernanda HernandezRoxana BohilteaFernando H. BartoloniRoxana RacoviceanuFernando Navarro GarciaRozenn RavallecFernando Padovan-NetoRuben ForestiFilippo BasagniRüdiger HehlmannFilippo Maria SantorelliRuei-Nian LiFilomena Anna DigilioRuey-Meei WuFiona CousinsRui ChenFiona SimpsonRui HenriqueFirdoos Ahmad GogryRui PereiraFiroz AhmedRui YanFiroz AkhterRuixue ZhuFityanul AkhyarRukmini MukherjeeFlavia FontanesiRumiana TzonevaFleming Martinez RodriguezRuna LindblomFlóra SzeriRune SlimestadFlorbela PereiraRuoxing WangFlorent LaferrièreRupak RajacharFlorentia FostiraRuth BroeringFlorian DuclotRyan PerkinsFlorian GesslerRyong Nam KimFlorian HeigwerRyszard AmarowiczFlorian M. WurmRyszard ŻabaFoteinos-Ioannis DimitrakopoulosSaad AlghamdiFotis BaltoumasSaad Mohammad AhsanFouquet GuillemetteSabata MartinoFrancesc Xavier AvilésSabina Antonela AntoniuFrancesca A. De GiorgiSabina PodlewskaFrancesca AndreettaSabine E. HammerFrancesca BorrelliSabino PachecoFrancesca BosciaSabira Mohammed JazirFrancesca BruzzeseSabrina BattistaFrancesca De FeliceSabrina Rahman ArchieFrancesca DuraturoSachchida Nand RaiFrancesca LonghenaSachin SarodeFrancesca MagheriniSadaf GilaniFrancesca MalagrinòSadeq VallianFrancesca ReggianiSaeid GhavamiFrancesca RiuzziSagarika BiswasFrancesca SantilliSaheem AhmadFrancesca UbertiSahil SharmaFrancesco BrunoSai LiFrancesco CiccareseSaid MougariFrancesco ClapsSaiful IslamFrancesco ClavicaSajal SenFrancesco Di GennaroSajjad AhmadFrancesco InchingoloSakellari DimitraFrancesco LeonettiSakineh Kazemi NoureiniFrancesco LiguoriSalah AmashehFrancesco LopezSaleem JavedFrancesco SianoSally Ann HuberFrancesco Tadini BuoninsegniSalva Reverentia YuristaFrancesco ZontaSalvador PérezFrancieli RohdenSam Mesiano MesianoFrancisco AmadoSamanta RaboniFrancisco Bianchetto-AguileraSamantha C. SalvageFrancisco ChavezSamantha GiordanoFrancisco Cruz-SosaSambandam RavikumarFrancisco J. BlancoSameera AbeyrathnaFrancisco J. NavarroSamir BarghoutFrancisco J. RoigSamiya Al-RobaiyFrancisco Jimenez-AcostaSamo LešnikFrancisco LeonSamuel BruFrancisco MesaSamuel J. LandryFrancisco NayaSamuel N. HeymanFrancisco Perez NevadoSamuel OjosnegrosFranck ChiappiniSamuel PitaFranck G. SturtzSandeep KumarFranck MartinSandeep SinghFranco VizeacoumarSandile Phinda SongcaFrancois FoulquierSandipan DattaFrank M. RaushelSandra BarbalhoFranka Klatte-SchulzSandra BarrosoFranz Felix KonenSandra BerndtFranz ZehentmayrSandra GhelardoniFranziska LauseckerSandra LeiszFrédéric EbsteinSandra LiebscherFrederic HeimSandra Martín-PeláezFrederic MelinSandra OrchardFrederike KramerSandra P. NunesFreeborn RwereSandra RistoriFriedrich AltmannSandra RocheFriedrich G. KappSandra SkujaFuai SunSandrine Cammas-MarionFuhua HaoSangbin LimFulong YuSangho RohFulvio D’AcquistoSang-Hwan HyunFulvio PupilliSangmin LeeFuming ZhangSang-Wook ParkFu-Yin HsuSanja DespotovićG Devanand VenkatasubbuSanming WangGábor BaloghSanti MandalGábor ErdösSantiago Ramon-MaiquesGábor TuruSantosh Kumar KunchaGabriel FenteanySara BaratchiGabriela Alejandra SalvadorSara LagoGabriela-Dumitrita StanciuSara Luz Morales-LázaroGadi TurgemanSara PahlavanGaël PanisSarah D’AlessandroGagan D. GuptaSarah HellewellGagandeep KaurSarah LummisGaia FaustiniSarah Santos GonçalvesGaia PellegriniSario GiannaGalen Andrew CollinsSarkar M. Abe KawsarGalia ZamaratskaiaSatheesh SengodanGanesh C. NikaljeSathish SankarGang ChenSatinder DahiyaGang DongSatya Murthy TadinadaGang ZhaoSatyavani KaliamurthiGarry KerchSaurabh ChattopadhyayGautam SethiSaurabh PandeyGea-Ny TsengSaveg YadavGeert J. SchenkSawan JalnapurkarGenjiro SuzukiSawsan ZaitoneGennaro EspositoSayaka MiuraGeno PeterScharfenberg FrankaGeoffrey Winston AbbottScott CanfieldGeorge Calin DindeleganScott I. SimonGeorge FthenakisSe Hun KangGeorge RoseSean BlamiresGeorge TherapondosSean ChiaGeorges MaestroniSebastian GranicaGeorgeta Violeta TruscaSebastián HormigoGeorgios Sokratis ChatzopoulosSebastian J. SchultheissGerald SeifertSebastian ZimmerGerard CagneySébastien BernacchiGerard DumancasSébastien PenninckxGerard M. TurinoSébastien Sébastien WieckowskiGerardo Vázquez-MarrufoSehyun ShinGerman Dominguez VíasSeley GharaneiGermán EbenspergerSeong Beom ChoGerman PerdomoSeon-Yong JeongGernot RiedelSepand RastegarGerrit BegemannSerena BestGerry LushingtonSerena LucottiGesmi MilcovichSerena VeschiGewei LianSergei A. MoshkovskiiGhulam Md AshrafSergei F. ChekmarevGiada MondanelliSergei GrudininGiampaolo MorcianoSergey SamsonovGian Luigi NicolosiSergio Rius-PérezGian Piero GalleranoSerguei KozlovGiandomenico AmicoSeung Gu ShinGianfranca CartaSeung-Heon ShinGianluca BaldanziSeung-Hwa KwakGianluigi MazzoccoliSeyed Khosrow TayebatiGianluigi VegliaSeyed Nasser OstadGilbert S. OmennSeyede Raheleh YousefiGill DiamondSeyun KimGina LisignoliSezai ErcisliGina-Cecilia PistolShagufta PerveenGiorgio LenazShagun KrishnaGiorgio TulliShahabaldin RezaniaGiorgos MarkouShailendra Kumar Dhar DwivediGiovanna De SimoneShailesh KhatriGiovanna GambarottaShaimaa SelimGiovanna LiguoriShakhawath HossainGiovanna MantiniShamas TabraizGiovanni AbatangeloShamima AkterGiovanni ButturiniShamir CassimGiovanni FilocamoShamroop Kumar MallelaGiovanni GalloShana StoddardGiovanni NanoShannon M. BaileyGiovanni PalleschiShantanu GuptaGiovanni PiccoliShaobin WangGiovanni ScarzelloShao-Hsuan KaoGiovanni StelitanoShaopeng PeiGiovanni VozziShaoyong LuGirish PattappaSharma NeelamGisselle N. MedinaSharmila GhoshGiuditta Dal CortivoShashank KumarGiulia GaggiShayne MasonGiulia RussoSheila SpadaGiulio VistoliShek Man ChimGiuseppe BenagianoSheng ZhangGiuseppe EspositoShengyun FangGiuseppe FiermonteSherry S. CollawnGiuseppe GrazianoShian-Ren LinGiuseppe LosurdoShiba DandpatGiuseppe MandraffinoShigenori TanakaGiuseppe RivaShigeo TakashimaGiuseppe ZanottiShigeori TakenakaGiuseppina AndreottiShiho NaitoGiuseppina NicoliniShiho OhnishiGiuseppina PalladiniShihua ZhangGiuseppina SabatinoShih-Yu HuangGladys AlexandreShin HamadaGlen E. KelloggShin MatsubaraGloria Álvarez-SolaShindu ThomasGloria Huerta-AngelesShingchern YouGloria IsaniShinichi AkanumaGomiero ChiaraShinichi HiranoGonçalo GarciaShinichi SakamotoGonzález AsierShinji MiyataGopinath M. SundaramShinobu SatoGoran GajskiShinya MatsuzakiGöran LarsonShira NinioGordana Wozniak-KnoppShireesh SrivastavaGorjana RackovShiri LiGottfried J. PalmShiv Dutt PurohitGraciela Caire JuveraShiv SrivastavaGrazyna GosciniakShiva HemmatiGreger LindbergShivanand PudakalakattiGregory C. HendersonShivangi InamdarGrigori RychkovShivani SathishGrigory V. ZyryanovShi-Zhen WangGrzegorz BartoszShizuka UchidaGrzegorz TrybekShobith RangappaGuang ShiShoji OdaGuangchun BaiShomona Gracia JacobGuangde JiangShooka MohammadiGuangyu WuShovan NaskarGuannan WangShravan GiradaGudrun KoehlShreyas D. GowdarGufa LinShubha Ranjan DuttaGuido MollShu-Chun ChangGuilherme Ribeiro RomualdoShuji OzakiGuillaume GrzychShujia ZhuGuillaume J. Van EysShu-Jui KuoGülçin TezcanShung-Te KaoGulsan Ara Sathi KaziShunichiro AsaharaGunavathi C.Shunji NatsukaGunnar KleinauShuo ChenGunnar WichmannShyam MoreGünter MüllerShyamala ThirunavukkarasuGünther SchmalzingShyamalava MazumdarGuodong CaoSi ChenGuohua LiSi WuGuojie WuSidney M. GospeGuoli HuSidney P. KuoGuowei WeiSieglinde ZelzerGuoxun ChenSiergiej M. DanilovGuozheng WangSigrid MillesGürdal SahinSijo MathewGurpreet Kaur AroraSileshi Gizachew WubshetGustavo FernandesSilva Katuŝić HećimovićGustavo GlusmanSilvano DragonieriGustavo Vieira De OliveiraSilvano GeremiaGuy RostokerSílvia A. SousaGyula TiminszkySilvia D’AgostinoHadi RastinSilvia Guzmán-BeltránHae-Seong NamSilvia LiskovaHai HuSílvia OsunaHaig Alexander EskandarianSilvia P. Tsanova-SavovaHaihong ShenSilvia RocchiccioliHaijun LiuSilvina LompardíaHaiqing ZhaoSilvio VeigaHaishu LinSima SepahvandHaiteng DengSimion AstileanHåkan AldskogiusSimon C. AndrewsHakim EchchannaouiSimon GutbierHakim Ouled-HaddouSimon T. AbramsHamilton CabralSimona FermaniHana PopelkaSimona SabbatiniHangkin KongSimona SerratìHan-Joo MaengSimone AnfossiHanna BaranowskaSimone BaldiHanna KmitaSimone Ciofi BaffoniHannu KoistinenSimone D. ScilabraHans BakkerSimone Di MiccoHanseob JeongSimone MirabiliiHansong MaSimone PersampieriHao Chun TsaiSimone SavinoHao HuangSimone TamburriHao TianSimone UmmarinoHao YangSimons SvirskisHaomin LiuSina NaserianHaoming ZhangSiniša D. GrozdanićHaopeng YangSinisa DjurasevicHaq Abdul ShaikSiniša MarkovHaragus HoriaSintu RongpipiHarald MischakSiqi HuHarant HannaSisitha JayasingheHardik KundariyaSitanshu S. SinghHarri LönnbergSiu Wai ChoiHaruhiko AsakawaSivakumar Vadivel GnanasundramHarvey SarnatSivaraman NatarajanHassen S. WolleboSjurdur F. OlsenHauke ThomsenSlađan PavlovićHayami ShinyaSladjana JevremovicHeba A. GadSławomir DreslerHector Riveros-RosasSlawomir GrabowskiHee Min YooSławomir MilewskiHeidi KidronSmriti PriyaHeidie Frisco-CabanosSnehal RautHeinrich JungSnježana MardešićHend M. El TayebiSobhi M. GomhaHendrik Rob TaalSoheb Anwar MohammedHeng YinSohsuke YamadaHenrich FrielinghausSoja SomanHenrik AnkersmitSokol TrunguHenrik G. DohlmanSolmaz MalekiHenrique FernandesSoňa ČiernikováHerman K. EdskesSónia Alves PereiraHerman SchreuderSonia CacciaHeui-Soo KimSonia FantoneHidekane YoshimuraSonja SielkerHideki HayashiSoochong KimHideki ShibataSoo-Ho ChoiHideki SugiiSoon Wook KwonHideki YokooSophie BriffaHideo WadaSophie C. PayneHidetaka TamuneSophie CaldérariHidetoshi KassaiSorel Sagu TchewonpiHiroaki TaniguchiSoumen DasHiroaki TsurumakiSoumya IyerHirofumi JonoSoumyadev SarkarHiroki AokiSounak SahuHiroki ToyodaSourav ChowdhuryHiromi SakaiSouvick RoyHiromi YanagisawaSpring R. FarrellHiromichi YumotoSravan Gopalkrishnashetty SreenivasmurthyHironobu AsaoSreenath NairHiroshi HandaSrinivas MutalikHiroshi KawakamiSrinivas SriramulaHiroshi KitagakiStacey SchutteHiroshi ManyaStane PajkHiroshi MatsuuraStanislav KotlyarovHiroshi NakagawaStanislaw OłdziejHiroshi SakaueStankevičius VaidotasHiroshi SugimotoStarling EmeraldHirotaka MiyamotoStefan HarsanyiHiroyoshi Y. TanakaStefan HoldenriederHiroyuki MiyachiStefan LiebauHiroyuki TsuchiyaStefan MihaicutaHiroyuki WakiguchiStefan SiwkoHisataka KobayashiStefania MerighiHisato YagiStefania OttavianiHisham FansaStefania PrincipeHishinuma ShigeruStefanie DopplerHo TsoiStefanie FlunkertHo Yeon KimStefanie MaurerHolger FlechsigStefano BorocciHolger StarkStefano CampanerHong JiangStefano Girolamo PalazzoloHong JinStefano SantabarbaraHong WanStefanos LeontopoulosHongjie WuSteffen GraetherHongmei MouStehmann ChristianeHongnan CaoSteliana GhibuHongqiao ZhangStephan BeiskenHongran YinStephan BlockHongyan LiStephan Joel ReshkinHongyang XuStephan SchremlHongzhu CuiStéphane BodinHosna JabbariStephane EsnautHossam Magdy BalahaStephanie BaudHossein Tabatabaei-JafariStephanie GoletzHsiang-Ruei LiaoStephen InbrajHsi-Chin WuStephen LewisHsien-Yuan LaneStephen R. BakerHsiu-Chung OuStéphen T. ManonHsiuying WangStergios BoussiosHsueh-Ju LuSteven A. BuechlerHua LiSteven J. FlieslerHuafeng XuSteven M. JohnsonHuai-An ChenSteven M. PascalHua-Jun FengSteven MullenHuanchen WangSteven WhittenHuanjiao Jenny ZhouStoyan PetkovHuashan ZhaoStylianos KorasidisHua-Sheng ChiuSu Datt LamHuaxiao Adam YangSubhradip KarmakarHuaxin ShengSubrat BhattamisraHugo Muñoz HernándezSudip PaulHui CaiSudip ShethHui-Hua HsiaoSudipto DasHuihui FanSue CotterillHuilan YueSue-Ann MokHui-lian ZhuSueli RodriguesHuiming LuSuhail AkhtarHun Hwan KimSuhrid BanskotaHunter MoseleySujeet KumarHwei Ling OngSuman SamantrayHye-Kyoung KimSumana GhoshHyemin LeeSumihiro MaedaHyouta HimenoSumonto MitraHyung Don RyooSunbin DengHyung-Goo KimSuncica BeluhanHyung-Kyoon ChoiSunčica RocaHyunil JoSung Ung KangHyunju LeeSunil K. SharmaIan M. C. DixonSunilgowda Sunnagatta NagarajaIan MorillaSunshin ChaIbrahim BoussaadSupriya JaggaIbrahim El-SayedSuresh ThanguduIfat SherSurya NauliIgnacio BejaranoSusana B. BravoIgnacio VasquezSusana Cohen-CoryIgor E. DeyevSusana CoimbraIgor SlivacSusanna CogoIhsan UllahSusanna NencettiIkhwan RinaldiSusanne SchulzIlaria BaglivoSusmitnarayan ChaudhuryIlaria FrassonSusumu MitsuyamaIlaria GuerrieroSusumu SuzukiIlaria OttonelliSutripto MajumderIlaria RivoltaSuzana ŠegotaIldiko RaczSuzuki YoshihiroIldiko SzantoSven-Erik BehrensIlias FrydasSvetlana DeryushevaIlias KappasSvetlana MishevaIlias MigdalisSvetlana SarantsevaIlias MylonisSvetlana SenikIlya UlasovSvetlana V. GusakovaIlze ŠtrumfaSwanand KoliIman TavassolySwapan ChakrabartyImran PanchaSwati SinghImre BakóSweta VangavetiIndra D. SahuSy Duong-QuyIndrajit SahuSydney B. MontesiIndu AmbudkarSyed Adeel HassanInes Vieira Da SilvaSyeda ShehzadiIngebjørg SeljeflotSylvia HsuIngo RustenbeckSylwia StudzińskaIngrida BalnyteSze Wan Samantha ShanInimary TobySzénási TiborInki MoonSzymon SękowskiInna ChabanT. Taskın-TokInna P. SolyanikovaT.J. HollingsworthInn-Kyu KangTabea SeeligerIoan Alexandru FlorianTadeusz KarczIoana E. PavelTae-Bong KangIoannis K. ToumpoulisTae-Jin KimIoannis KanakisTaha Koray ŞahınIoannis LiampasTahereh JavaheriIoannis MichalopoulosTahmineh MokhtariIoannis PetrakisTaisuke SeikeIogann TolbatovTai-Yi JinIoly Kotta-LoizouTakafumi TsuboiIonut LuchianTakahiro BizenjimaIraide AllozaTakahiro HorieIrena MajerzTakahiro NagatakeIrena NalepaTakashi NakakukiIrena RotermanTakayuki MurataIrene Díaz-MorenoTakayuki NagaiIrene PaternitiTakehito OuchiIrene Russo KraussTakemichi FukasawaIrfan KhanTakeshi KajiharaIrina D. KonstantinovaTakeshi KawauchiIrina EliseevaTakeshi UemuraIrina KondakovaTakumi UedaIrina-Georgeta SufaruTakuya TadaIris RibitschTalha Mahboob AlamIris SilvaTamara MerzIsaac Uzoma AsuzuTamás HofmannIsabel CasimiroTamás JuhászIsabel MarzoTamas LazarIsabel Oroz-GuineaTamas MarosvolgyiIsabel Soto-CruzTamás MicsikIsabelle CallebautTanja J. MiličićIsabelle ChantretTanmoy SanyalIsela Montúfar-RoblesTao HuangI-Shiang TzengTao ZengIsrael SilmanTao ZhangIstván SzokodiTarsila G. CastroItai BenharTarun AgarwalItzel López-RosasTasadduq ImamIurii S. StafeevTateaki NaitoIvaldo ItabaianaTatiana BetakovaIvan BassaniniTatiana I. ArefievaIvan GiovanniniTatiana M. VinogradovaIvan KreftTatiana MatveevaIván Martínez-ValbuenaTatiana RameyIvan SalarićTatiana ScorzaIvana GiangriecoTatiana WojciechowiczIvana ProdićTatiana Yu KuznetsovaIvana StojanovicTatjana ĆosićIvana V. SofrenićTatjana RadosavljevićIveta BernátováTatsuya AraiIveta WaczulíkováTatsuya MoriyamaIwona BryndalTatyana DanyukovaIwona PuzioTatyana P. ShakhtshneiderIwona RzeszutekTatyana PolenovaIwona SaduraTatyana StrekalovaIzabela DudaTaulant MukaIzabela KruszelnicaTawanda ZiningaIzumi HorikawaTeh-Min HuIzumi OritaTejeshwar RaoIzuru KawamuraTeka KhanJ. Arturo Garcia HorsmanTelma QuintelaJ. C. Luis JorgeTeodoro Coba De La PeñaJacek NyczTeresa Hernández-SotomayorJacek WalukTeresa PinheiroJackeline FrancoTerry C. JonesJacopo IacovacciTerry GentryJacopo Junio Valerio BrancaTetsuhiro KudohJacqueline BarlowTetsuya TakahashiJacques. ObertoThaigarajan ParumasivamJae Boum YoumThammarat AreeJae-Hyeok LeeTheodoros I. RoumeliotisJae-min ChaTheodoros KaralisJaewhan SongTheoni MargaritopoulouJafar RezaieTheophilos TzaridisJagodnik KathleenThibault ViennetJakub KaminskyThierno Madjou BahJamal BouitbirThierry HocJames A. MarrsThierry PrangéJames B AmesThirumaran ThanabaluJames ButcherThomas ArzbergerJames E. Jr. HassellThomas BottonJames M. GruschusThomas D. GossiosJames P. BennettThomas F. MooreJames R. LupskiThomas FriedmanJames StewartThomas J. OrtonJames T. Van LeuvenThomas KeanJan BocianowskiThomas KloseJan E. AzarovThomas MarshJan GebauerThomas MehnerJan Gettemans GettemansThomas MohrJan KotekThomas TschernigJan L. MiljkovićThorsten AllersJán LehotskýTiago Campos PereiraJán LeštákTianhui HuJan NovákTibor HortobágyiJan PetrTigran ChalikianJan StrnadelTilahun Ayane DebeleJan Van Den BosscheTilman SchlothauerJan ZabrzynskiTimea SpakovaJana MašlankováTimo SorsaJana Sopková-De Oliveira SantosTimothy EubankJanakiram R. VangalaTimothy GreenJani SilvaTimothy HuangJanja ZupanTimothy James MottramJanusz GodlewskiTimothy John MahonyJara Pérez-JiménezTimothy M. ReynoldsJardel Francisco Mazzi-ChavesTimothy McCubbinJared T. AhrendsenTimothy MeadJasenka ZubcevicTin NguyenJasmin KnopfTina Tičinović KurirJasminka TalapkoTing ZhongJason BrayerTingxiang YanJason HannaTing-You WangJason T.C. TzenTiphaine MartinJason Thomas DuskeyTirumala Kumar ChowdaryJavad HadianTiziana AlberioJavier Leon SerranoTiziana CollettiJavier Martínez TorresTodd E. ScheetzJavier NavalTodd EckroatJay AnandTodd FiaccoJay H. ChungTofazzal IslamJay TrivediTom TurkJean Christophe RouxTomasz CzapikJean Christopher ChamcheuTomasz FrączykJean GalitzkyTomasz KowalczykJean M. F. CustodioTomasz LitwinJean Marie BillardTomasz RogozińskiJean-Claude MartinouTomasz SekutowskiJean-François QuignardTomasz StompórJean-Marc BarretTomasz TrombikJean-Marie RuysschaertTomasz UrbanowiczJean-SéBastien AnnicotteTomasz WentaJed LampeTomasz ZielinskiJee-Ching WangTommaso PiccoliJeevithan ElangoTomoharu TakeuchiJeffrey L. DupreeTomohide SaioJeffrey M. WilsonTomohiro MizobataJeffrey StuartTomoki MaekawaJeffrey V. LeytonTomoko AokiJeffrey WickliffeTomoya FujieJehan AlamTomoyuki IwataJelena MijalkovićTonali Blanco AyalaJelena VladićTong-Hong WangJelica LazarevicTorben RedmerJen-Chieh TsaiToru ImamuraJenni Johanna HakkarainenToshiaki NakanoJennifer FazzariToshiyuki MatsuzakiJennifer GebetsbergerToyoaki OhbuchiJennifer S. EsserTracey NewmanJenny DesantisTracy Murray-StewartJens KockskämperTran Dang KhanhJeong A. ParkTran Dang XuanJeong HeoTrayambak BasakJeong Hwan LeeTripti JoshiJeong-Yeon LeeTristan Chalvon-DemersayJeppe PrætoriusTroy HornbergerJeremie D. OliverTruls E. Bjerklund JohansenJérémie RossyTsehay Admassu AssegieJeremy TurnbullTsun-Thai ChaiJerome DevyTsuyoshi ShiraiJérôme RoyTuerdimaimaiti AbudulaJer-Yiing HoungTuo MengJerzy ChudekTuoen LiuJessica P. AnandTyler J. TitcombJessica RosatiTzu-Chieh TangJesus Perez-LosadaTzung-Han ChouJetze TepeUgo BastollaJi WangUgo CarraroJia LiuUjendra KumarJia XiongUlf DettmerJiachen HuangUlises De La Cruz-MossoJiacun WangUlises Gomez-PinedoJiajia SongUlrich EckhardJiali WangUlrich JuliusJialong ShenUlrich SchörkenJian LiUlrike BacherJian SangUmberto DianzaniJian WangUmberto MalapelleJian YinUmesh R. DesaiJian ZuoUrminder SinghJianfeng LiUrsula FleigJiang-Yun LuoUtpal DasJianli DuanUttam BhattaraiJianling XieV.G. VaidyanathanJianping WuVadanasundari VedarethinamJianqiang XuVadim KumeikoJianxin WangVadim SumbayevJianzhou CuiVadim TseilikmanJiaqi MiVahideh RabaniJiayu ZhuValentin NastasaJibin ZhangValentina FragliassoJie DongValentina KovalevaJie YangValentina PozziJie ZhuangValentina TostoJihan XiaValentina VairaJihua GuoValentina VassalloJiiang-Huei JengValentine VullevJikui GuanValentyn OksenychJillian MadineValeria ContiJi-Long LiuValeria Di DatoJin WangValeria MengaJindong XieValeria MericoJindrich CitekValeria PanzettaJing HeValeria ScalaJing JinValérie SamouillanJing LuoValerio FerrarioJing YangValerio FulciJing-Hua YangValter LubranoJingli XieValter TravagliJingrui ChenVanesa GottifrediJing-Song OuVanessa DehennautJingwen CaiVânia Vilas-BoasJingwen YangVartika SharmaJingxia LiuVarun MaturiJingya LiVasil AtanasovJinhae KimVasileios SiokasJin-Jia HuVasiliki TsigkouJinling HuangVasilios E PapaioannouJinming HanVasily SukhorukovJinrong MaVassilis KarageorgiouJiri PetrakVelmarini VasquezJiro TakitoVenkata Rajesh YellaJishnu KrishnanVenkata Reddy ChirasaniJitlada VichapongVenkata SabbasaniJiuann-Huey Ivy LinVera A. KrischikJiucun WangVera M. ChesnokovaJi-Yong Julie KimVerica MilivojevicJJ LuszczkiVeronica Alexandra AntipovaJo Anne StrattonVeronica PalmaJoan AlbiolVerónica Rocío Vásquez-GarzónJoan OlivaVeronica SteriJoanna BieleckaVerónica Zaga-ClavellinaJoanna BierłaVeronika BrezovakovaJoanna BogusławskaVeronique BonnetJoanna FilipowskaVíctor García-GonzálezJoanna Gdula-ArgasińskaVictor I. TsetlinJoanna KozakVictor LucioJoanna ListosVictor M Bolanos-GarciaJoanna SikoraVictor MaltsevJoão Gaspar-MarquesVictor MarchenkovJoao R. GomesVictor MatveevJoão Ramalho-SantosVictor PleshkanJoão VicenteVictoria Ramírez GonzálezJoaquim JaumotVictoria RoshchinaJoaquim PinheiroVictoria VitkovaJoaquin De NavascuesVictor-Vlad CostanJoaquin Manzo-MerinoVidal Delgado RizoJocelyn InamoViera SchwarzbacherováJocelyne DiRuggieroVijay Kumar SiripuramJochen SchaeferVikas KushwahaJoëlle WielsVikram JoshiJoerg ReindersViktoria RufJohan RenesViljem VekJohann SchredelsekerVimal ChadhaJohannes FesslerVincent PonsJohannes HoferVincenzo FiorentinoJohannes SchlondorffVincenzo QuagliarielloJohannes Von LintigVineet K. GuptaJohn CuppolettiVineeta RaiJohn E. PiletzVinod RaviJohn E. PintarVinod VijayakurupJohn E. SchjenkenVirginia TzankovaJohn J. TurchiVirginie DoceulJohn KruifVishal B. SiramshettyJohn Man Tak ChuVishal K ChavdaJohn RudanVita DolžanJohn S. DavisVito De PintoJohn S. OlsonVitor OliveiraJohn T. ConnellyVittorio CalabreseJohn TsiligkaridisVittorio PicchioJohn W FroehlichVivek ChoudharyJohn ZaundersVivek DharwalJohnannes Jacob De SoetViviana Valadez-GrahamJohn-Paul PlazzerVladiana TuriJohnson V JohnVladimir ChubanovJolanta WierzbaVladimir GureevJon Del ArcoVladimir Mulens-AriasJonathan A LindquistVladimir MuronetzJonathan BallVladimir P. ZorinJonathan N. FlakVladimir PalyulinJong Han LeeVolkhard HelmsJong-Eun KimVui King Vincent-ChongJongmin KimVyron GorgogietasJoo Ho LeeWael A. KhalilJoongkyu ParkWael HegazyJorddy Neves CruzWalaa NegmJordi OliverWaldo CerpaJörg GsponerWalee ChamulitratJörg ReichenwallnerWalter SandtnerJorge A. MoralesWalter SerraJorge A. PalermoWang QiJorge CuéllarWaqas WakilJorge Gonzalez-GarciaWaruna DissanayakaJorge Jorge SimonWataru NishimuraJorge PeixinhoWei Guang SeetohJorge TrillerasWei GuoJosé Ángel ArmengolWei Wen LimJosé António CoutoWei ZhengJosé Antonio Morales-GonzálezWei ZhongJose Antonio Pariente LlanosWeichih ChenJose BaezaWei-Dong YaoJosé Cleiton Sousa Dos SantosWeihao YuanJosé Daniel CaminoWeihong LuJosé EscarioWei-hsiung YangJose Felix. Moruno ManchonWeikuan GuJosé Francisco IslasWeikun XiaoJose G. CastanoWeiwei XueJose Gomez-SanchezWeiyi HeJosé Luis Pérez-CastrillónWenbo YuJose M. RojoWenbo ZhiJosé Miguel Seguí-RipollWendel SilvestreJosé P. Cerón-CarrascoWenguang LiJosé Pedraza ChaverriWenhua ZhengJosé Rafael De AlmeidaWenjun XieJose Rodriguez-AlvarezWenrui HaoJose Sanchez-AlcazarWen-Ta SuJose TudelaWen-Tsong HsiehJosef JampilekWenxia WangJosep M. Cambra BortWenyi FengJoseph C. AndersonWenyu WenJoseph M. RosenWijeratne AselaJoseph PapamatheakisWiley BartonJosh HihathWilliam BlalockJoshua LevyWilliam GriffithsJoshua S. WallaceWilliam SchwanJosiah OchiengWilliam W. LyttonJosko BozicWilson Chun Yu LauJosué V. Espinosa-JuárezWinfried M.K. AmoakuJosy AugustineWioletta PawlukowskaJoy MitraWisit KaewputJože GrdadolnikWładysław LasońJózsef JászaiWojciech KruszynskiJuan Bautista De SanctisWolfgang FischerJuan C. AlmagroWolfram S. KunzJuan M. GonzalezWon Hyuk SuhJuan Manuel Guzmán-FloresWonhyo SeoJuan NavarroWoojin KangJuan Rodrigo SalazarWoong Sub ByunJuan RosadoWoo-Yoon ParkJudit OláhWuyi MingJudith DavieXanthopoulos KonstantinosJudith PetersXaolin ChenJuei-Tang ChengXavier ThuruJu-Fang LiuXavier Vidal-GómezJulia ChisholmXiakun ChuJulia FuchsXiang WangJulia Sanchez-CeinosXiangbing MengJulián AragonésXiang-Guo LiJulian FriebelXiangjun ChenJulián Rodriguez TalouXiangli ZhaoJuliana De Oliveira SilvaXiangming JiJuliano UczayXiangxi MengJulien SebagXiao LinJulio MoralesXiao XuJulius RablXiaochen HeJun HamazakiXiaodong WangJun InoueXiaogang ZhangJun ItamiXiaogen ZhouJun NakaeXiaojie LiuJun NishikawaXiaolei TangJun OhnoXiaoliang QiJun SugimotoXiaoliang WangJun Sung MoonXiaolong ChenJun ZhouXiaoming FangJunaidi KhotibXiaoyin YangJunchao TongXiaoyu DingJung Y. HuangXiaoyu SongJung-Ho ShinXiming GuoJung-Ho YoonXin Jie ChenJunhui PengXin SunJunji FukudaXincheng YaoJunji YamauchiXinda LiJunxia LuXingang GuanJun-Xiang BaoXinrong GuoJunya ItoXinyi LiJunyang JungXisheng WengJuraj PiestanskyXiuqiong BiJürgen BehrensXixi ZhouJürgen KreiterXuan MeiJustas DapkūnasXuan ZhangJustyna MaliszewskaXudong LiJustyna StrycharzXuefeng DuanJW Cohen TervaertXuehua XuJya-Wei ChengXupeng HongJyh-Fei LiaoXuwen LiJyh-Yeuan LeeYafeng LiK. KisandYajie GuKa Yee FungYajun XieKah Hui WongYan HuangKai SunYan LeeKai YuYan ShuKaihui LuYan WangKaito TakahashiYan XiongKaiyang ZhangYan ZhuKai-Yuan LinYana IgolkinaKalaivani ParamasivanYang JiangKalyan Sundar GhoshYang LiuKamal Al NasrYang YangKamalika MojumdarYang ZhangKamil GrubczakYang ZhaoKamil KaminskiYang ZhouKamil KostynYangpeiwei HuangKamilla SwiechYanjun ZhangKamlesh BhopaleYanna CaoKamyar ZahediYanni Ka-Yan ChinKanji TsuruYannick RossezKanwal RehmanYannis KaramanosKanwarpal SinghYantao LiangKaoru YoshidaYanxian LinKar Wey YongYasir IqbalKaram El-BayoumyYasuhiko KizukaKaranovic DanijelaYasuhiro KogaKarel AlcedoYasuhiro TsujiKarel SmetanaYasuhito IshigakiKaren ChristensenYasunari MatsuzakaKaren Vivien LithgowYasushi YabukiKari L. StoneYasushige ShinguKarima SchwabYau Kei ChanKarin BuschYa-Xiong TaoKarina Gonzales SilverioYe LiuKarina JouravlevaYelena V. ParfyonovaKarina Kubiak-OssowskaYen-Ying KungKarl-Friedrich BeckYeon Gyu YuKarolina A. Wojtunik-KuleszaYichao LiKarolina DziemidowiczYichen LiKarolina SzczepanowskaYi-Cheng ChenKarrer M. AlghazaliYifeng QiKarsten-Henrich WeylandtYih-Fan ChenKarthikeyan MuthusamyYilai ShuKarunakar Reddy PothulaYilin LiuKatalin F. MedzihradszkyYimin HuangKatarína ŠipošováYinan JiangKatarina VukojevicYing YangKatarzyna A. NałęczYingJu LaiKatarzyna BłochowiakYingjun LiuKatarzyna FischerYi-Ting WangKatarzyna Komosinska-VassevYiwang ZhouKatarzyna LewandowskaYiwen MengKatarzyna Pawińska-WąsikowskaYixing DuKatarzyna PiórkowskaYogesh GoyalKatarzyna ZdanowiczYohannes ShiferawKateřina BišováYohei OkadaKatharina A. ScherfYoichiro AbeKatharina DuliasYolanda García-MesaKatharina HöferYolanda SalinasKatharina RumpYong ChenKatharina ScherschelYong LongKatherina SiewertYong Mee ChoKatherine R. HixonYong ZhouKathleen L. HefferonYongguo LiKatiuska SatuéYongkang YangKatja SeipelYong-Pil CheonKatrijn R. SixYoon Ki KimKatsuhiko MurakiYoon Young KimKatsuhiko TabuchiYoonjee ChaeKatsuki TsuchiyamaYoori KimKatsumasa GotoYorito HattoriKatsunori YoshidaYork MarahrensKatsutoshi YayamaYosef ShaulKatsuya HirasakaYoshiaki ZaizenKaty OvingtonYoshiharu KimuraKavita SharmaYoshihiko ItoKaviya ChinnappaYoshihiro KomatsuKazi Lutful KabirYoshihiro ShidojiKazuei IgarashiYoshimasa AsoKazuhide Shaun OkudaYoshinori InagakiKazuhiko KibayashiYoshiro ChumanKazuhiro ShigemotoYoshiro SuzukiKazuhito SuzukiYoshitaka HosokawaKazunori SangoYoshito TsushimaKazuto TajiriYoshiya OdaKazuyuki NakagomeYoshiyuki NakamuraKe XiaoYoshiyuki NishimiyaKeigi FujiwaraYosuke YamawakiKeiichiro InamoriYotam LiorKeiji KomatsuYoungjoo KwonKeiji TanimotoYoung-Jun ParkKeiko HosohataYoung-Sook KangKeith MorrisYoussra Al-HilalyKelly M. SchultzYou-Zhi TangKelvin G. M. BrockbankYsander Von BoxbergKen TakasawaYu SunKengo SatoYu Ting TsaiKênia Pedrosa NunesYu XuKenichi YamadaYu ZhangKen-ichiro MinatoYu ZhaoKening LangYu-An HuangKenneth I. OnyedibeYuan-Shuo HsuehKenneth S. ShindlerYub Raj NeupaneKentaro InoueYuemin BianKentaro TomiiYuexiu ZhangKermit L. CarrawayYufeng ZhaoKeshari ThakaliYugo AshinoKe-Vin ChangYuhan HaoKevin FreedmanYuichi MatsuuraKevin KennaYuichi TsuchiyaKevin MolloyYuichiro WatanabeKevin WelsherYuji NagatomoKhadija El HadriYuji ShimizuKhajamohiddin SyedYukihiko MomiyamaKhaled El-TarabilyYulia Yu StroylovaKhaled MachacaYu-Lin HsiehKhalid RazaYuliya I. RaginoKhalil KarimiYuliya S. KrasikovaKhushwant Singh BhullarYun ShiKiersten M. RuffYun Wah LamKilto ChongYunfeng HuangKim TieuYung-Hsiang ChenKimberly JasmerYuntao LiuKinga NyíriYuri PersidskyKira V. VyatkinaYurong LiangKiraly LaszloYury A. ErmakovKirill AntonetsYury KistenevKirsten Haastert-TaliniYusuke KatoKiyofumi TakabatakeYutao LiuKiyonobu HommaYves BoucherKlaus FortscheggerYves MechulamKlaus PetryYvonne LiKlaus WimmersZachary SmithKoh FujinagaZahra AshkavandKohei UchimuraZanda BakaevaKohei YamaharaZanmin HuKoichi FurukawaZanxia CaoKoichi HonkeZbigniew J. WitczakKoichi KitagawaZehuan LiaoKoichi NaitoZeljko SvedruzicKoji MiyamotoZengqian ShiKoji NishimuraZeqing ChenKoji TamuraZhangjian ChenKoldo Garcia-EtxebarriaZhan-Guo GaoKomuraiah MyakalaZhaojia GeKonda Mani SaravananZhaoqian TengKonda Srinivasa RaoZhaoxi SunKonstantin BelosludtsevZhe YangKonstantin VolchoZhen ShuKonstantinos TziomalosZhen WangKookhui RyuZhenbo HanKornél KirályZhenbo TuKoshi AkahaneZheng FuKripa ShankarZhengxiang HuangKrishna Chaitanya PavaniZhenhua ShaoKrishna ChinthalapudiZhenxin WangKrishna Mohan Padmanabha DasZheqi LiKrishna PersaudZhi YueKrishna SharmaZhidong ZhouKrishna ShrinivasZhihong YangKrishnan RathinasamyZhijie QiKrishnendu PalZhili ZuoKristaps JaudzemsZhiliang JiKristel MijnendonckxZhi-Ren LiuKristen S. HillZhiyuan LiKristin SurmannZhongtian LiuKristine GriffettZhulin HuangKristóf Endre KárolyZichao LuoKristoffer PeterssonZicong LiKrisztián KvellZijian ZhangKrisztián PajerZili XieKrisztina VargaZiliotto SilviaKrithika RajagopalanZiping ChenKrystyna CwiklinskiZiyad S. HaidarKrzysztof DołowyZongjin LiKrzysztof Durkalec-MichalskiZoran IvanovicKrzysztof GorynskiZozan GulekenKrzysztof JaglaZsolt BacsoKrzysztof SztankeZsolt GallaKrzysztof WięckowskiZsolt SarangKshitiz KshitizZsuzsa BagolyKuldeep MakwanaZufang WuKulthida VaeteewoottacharnZulqurnain SabirKumar Chandan SrivastavaZu-Quan HuKumar GanesanZygmunt Warzecha


